# Quantum-criticality-induced strong Kerr nonlinearities in optomechanical systems

**DOI:** 10.1038/srep02943

**Published:** 2013-10-15

**Authors:** Xin-You Lü, Wei-Min Zhang, Sahel Ashhab, Ying Wu, Franco Nori

**Affiliations:** 1 CEMS, RIKEN, Saitama, 351-0198, Japan; 2 Wuhan National Laboratory for Optoelectronics and School of Physics, Huazhong University of Science and Technology, Wuhan 430074, People's Republic of China; 3 Department of Physics, National Cheng Kung University, Tainan 70101, Taiwan; 4 Physics Department, The University of Michigan, Ann Arbor, Michigan 48109-1040, USA; 5 Department of Physics, Korea University, Seoul 136-713, Republic of Korea

## Abstract

We investigate a hybrid electro-optomechanical system that allows us to realize controllable strong Kerr nonlinearities even in the weak-coupling regime. We show that when the controllable electromechanical subsystem is close to its quantum critical point, strong photon-photon interactions can be generated by adjusting the intensity (or frequency) of the microwave driving field. Nonlinear optical phenomena, such as the appearance of the photon blockade and the generation of nonclassical states (e.g., Schrödinger cat states), are demonstrated in the weak-coupling regime, making the observation of strong Kerr nonlinearities feasible with currently available optomechanical technology.

Strong optical nonlinearity gives rise to many important quantum effects, such as photon blockade^1^,^2^,^3^, quantum squeezing^4^, quantum nondemolition measurements^5^,^6^, optical switching with single photon^7^ and so on^8^,^9^,^10^. These nonlinear optical effects have been demonstrated in cavity QED systems, where the quantum coherence in the atom^1^,^2^,^3^ (or artificial atom^11^,^12^,^13^,^14^,^15^,^16^,^17^) generates strong effective photon nonlinearities.

Recently, cavity optomechanics has become a rapidly developing research field exploring nonlinear coupling via radiation pressure between the electromagnetic and mechanical systems^18^,^19^,^20^. It has been shown *theoretically* that strong optical nonlinear effects (and relevant applications, such as generating nonclassical state, photon blockade, multiple sidebands, photon-phonon transistors, and optomechanical photon measurement) can be realized in single-mode^21^,^22^,^23^,^24^,^25^,^26^,^27^,^28^,^29^,^30^,^31^,^32^,^33^ or two-mode optomechanical systems (OMSs)^34^,^35^. These phenomena are mainly demonstrated in the single-photon strong-coupling regime, where the optomechanical coupling strength at the single-photon level *g_a_* exceeds the cavity decay rate *κ_a_* (*g_a_* > *κ_a_*). However, in most experiments to date^36^,^37^,^38^, *g_a_* is much smaller than *κ_a_* (*g_a_*/*κ_a_* ~ 10^−3^). Only a few new-type optomechanical setups, using ultracold atoms in optical resonators (*g_a_*/*κ_a_* ~ 10^−1^)^39^ or optomechanical crystals (*g_a_*/*κ_a_* ~ 10^−2^)^40^, can one begin to approach the single-photon strong-coupling regime. On the other hand, a strong optical driving field may effectively enhance the optomechanical coupling by a factor 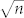, where *n* is the mean photon number in the cavity^41^,^42^,^43^. But this enhancement comes at the cost of losing the nonlinearity of the interactions. Specifically, under the condition of strong optical driving, the linearized coupling strength between the optical and mechanical modes is largely enhanced, which makes the intrinsic nonlinear optomechanical coupling smaller and negligible.

Given the above, it is highly desirable to find a new method for obtaining strong Kerr nonlinearities in OMSs in the *weak-coupling regime*, namely the optomechanical coupling strength is much smaller than the optical cavity decay rate (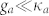). In this paper, we investigate the Kerr nonlinear effects of the optical field in a hybrid electro-optomechanical system containing a mechanical oscillator coupled to both an optical cavity and a microwave *LC* resonator (see Fig. 1)^44^,^45^,^46^,^47^. We find that the eletromechanical subsystem (the mechanical oscillator plus the microwave resonator) displays a quantum criticality. One can drive the electromechanical subsystem close to the quantum critical regime by applying a microwave field with properly chosen frequency and intensity to the microwave resonator. Then the quantum criticality can induce a strong Kerr nonlinearity in the optical cavity, even if the optomechanical systems (the optical cavity and mechanical oscillator) is in the weak-coupling regime. This strong Kerr nonlinearity can be demonstrated by the existences of photon blockade and nonclassical states (e.g., Schrödinger cat states) of the cavity field when the electromechanical subsystem approaches the quantum critical point. Furthermore, the strong Kerr nonlinearity can also be controlled easily by tuning the intensity (or frequency) of the microwave driving field. This provides a promising route for experimentally observing strong Kerr nonlinearities in OMSs in the weak-coupling regime.

## Results

### Hybrid electro-optomechanical system

**Figure 1:**
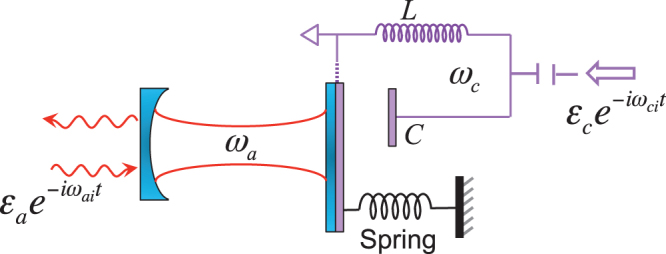
Schematic diagram of the hybrid electro-optomechanical system. A mechanical oscillator couples to both an optical cavity and a microwave *LC* resonator.

In the hybrid electro-optomechanical system of Fig. 1, the mechanical oscillator is parametrically coupled to both the optical cavity and the microwave resonator. The microwave resonator is driven by a strong field with amplitude *ε_c_* and frequency *ω_ci_*, where *ε_c_* is related to the input microwave power *P* and microwave decay rate *κ_c_* by 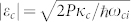. In a frame rotating with frequency *ω_ci_*, the Hamiltonian for this hybrid systems reads^48^

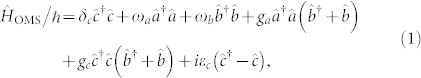
where the detuning *δ_c_* = *ω_c_* − *ω_ci_* and the microwave frequency 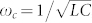, *g_a_* (*g_c_*) denotes the optomechanical (electromechanical) coupling strength at the single-photon level, and 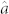 (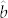 or 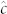) is the annihilation operator of the optical cavity (the mechanical oscillator or the microwave resonator). Under a strong microwave driving field, following the standard linearization procedure^49^,^50^,^51^,^52^ (shifting 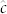 and 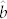 with their steady-state mean values *α* and *β*, i.e., 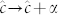, 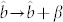), the Hamiltonian can be transformed into 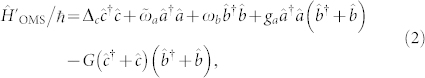
where *G* is the linearized electromechanical coupling strength; Δ*_c_* and 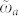 are, respectively, the effective microwave detuning and optical frequency including the radiation-pressure-induced optical resonance shift. Their explicit expressions are given by 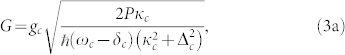

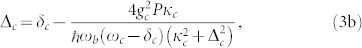

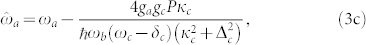
Notice that *G* and Δ*_c_* can be easily controlled by tuning the power and frequency of the microwave driving field.

### Quantum critical property of the electromechanical subsystem

The quantum criticality in the electromechanical subsystem can be shown by diagonalizing the electromechanical subsystem via a Bogoliubov transformation 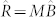. Here, the canonical operators are 
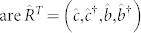
 and 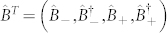, and M is the transformation matrix given by 
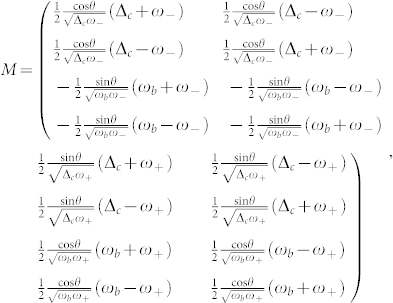
where the angle *θ* is defined by 
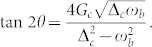


**Figure 2:**
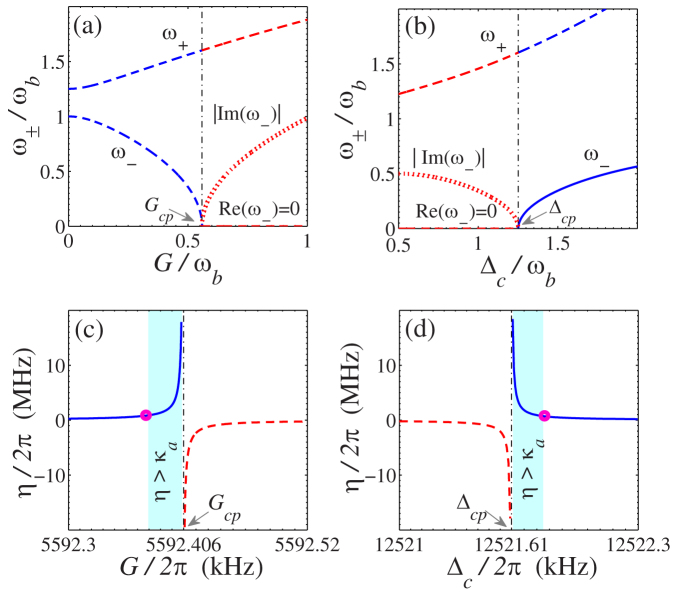
Quantum criticality of the electromechanical subsystem and strong Kerr nonlinearity of the optical field. (a,b) Quantum criticality of the electromechanical subsystem, characterized by the normal-mode frequency *ω*_±_/*ω_b_*. As one can see, the normal-mode *ω*_+_ continuously passes through the critical point. The quantum criticality is manifested with the normal-mode *ω*_−_, which becomes purely imaginary after the critical point *G*/*ω_b_* > 0.5 and Δ*_c_*/*ω_b_* < 1.25 (c,d) Strong Kerr-nonlinearity given by the photon-photon interaction strength *η* in the optical cavity, as a function of the adjustable parameters *G* and Δ*_c_* controlled by the microwave driving field. The pink circles and shaded area in (c,d) correspond, respectively, to the regimes *η* = *κ_a_* and *η* > *κ_a_*. The black dot-dashed vertical lines indicate the quantum critical points *G_cp_* and Δ*_cp_*. Other system parameters are taken as: *ω_b_*/2*π* = 10 MHz, *g_a_*/*ω_b_* = *g_c_*/*ω_b_* = 10^−3^, *κ_a_*/*ω_b_* = 0.1, *κ_c_*/*ω_b_* = 0.127, Δ*_c_*/*ω_b_* = 1.251 (a,c), and *G*/*ω_b_* = 0.5595 (b,d).

Then, the Hamiltonian 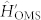 becomes 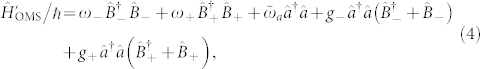
where *ω*_±_ are the normal mode frequencies of the electromechanical subsystem, 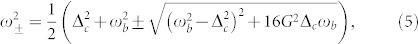
and 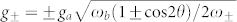
are the effective coupling strengths between the optical photon and the normal modes. Equation (5) shows that 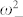 becomes zero (negative) when 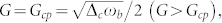
as shown in Fig. 2(a). This corresponds to a critical property^53^, namely, the normal mode *ω*_−_ will change from a standard harmonic oscillator (*G* < *G_cp_*) to a free particle, and further becomes dynamically unstable (*G* > *G_cp_*) as *G* crosses its critical value *G_cp_*.

**Figure 3:**
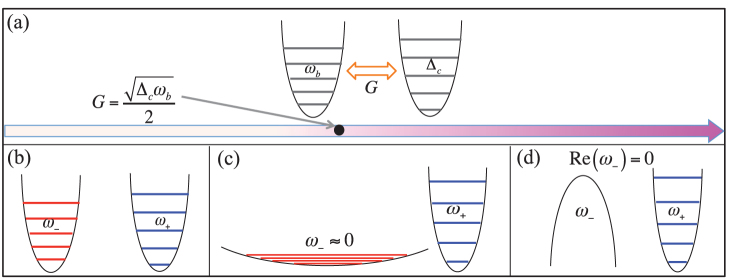
The critical property of the electromechanical subsystem. (a) The mechanical and electrical modes couple with each other with the coupling strength *G*. The black circle indicate the quantum critical point. (b,c,d) The effective potential of the normal mode *ω*_−_ becomes flat and further inverted when increasing the coupling strength *G*.

The above critical property can become more transparent with the following canonical relationships: 
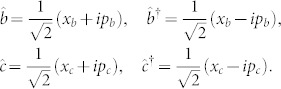


Here *x_b_*, *x_c_* are the dimensionless displacements of the mechanical and microwave resonators from their stable points, and *p_b_*, *p_c_* are the corresponding dimensionless momentums. The Hamiltonian of the electromechanical system can then be written in terms of the usual canonical *x*–*p* variables, *H*_e–m_ = *H*_0_ + *H*_int_ with 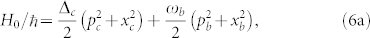

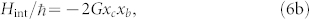
denoting the free Hamiltonian of the microwave and the mechanical resonators, and the coupling between them. The potential of the free Hamiltonian (6a) can be further expressed as 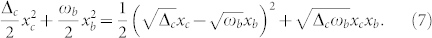
It shows that the intrinsic potential of the electro and mechanical resonators is characterized by 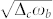. Comparing Eq. (7) with the coupling Hamiltonian (6b), one can see that there is an interplay between the intrinsic potential and the coupling interaction between them. This interplay leads to the above critical property. In other words, when *G* approaches (or exceeds) 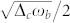, the normal mode *ω*_−_ is dragged out from its effective potential, and becomes increasingly flat (or inverted) [see the Fig. 3].

### Quantum-criticality-induced strong Kerr nonlinearities

**Figure 4:**
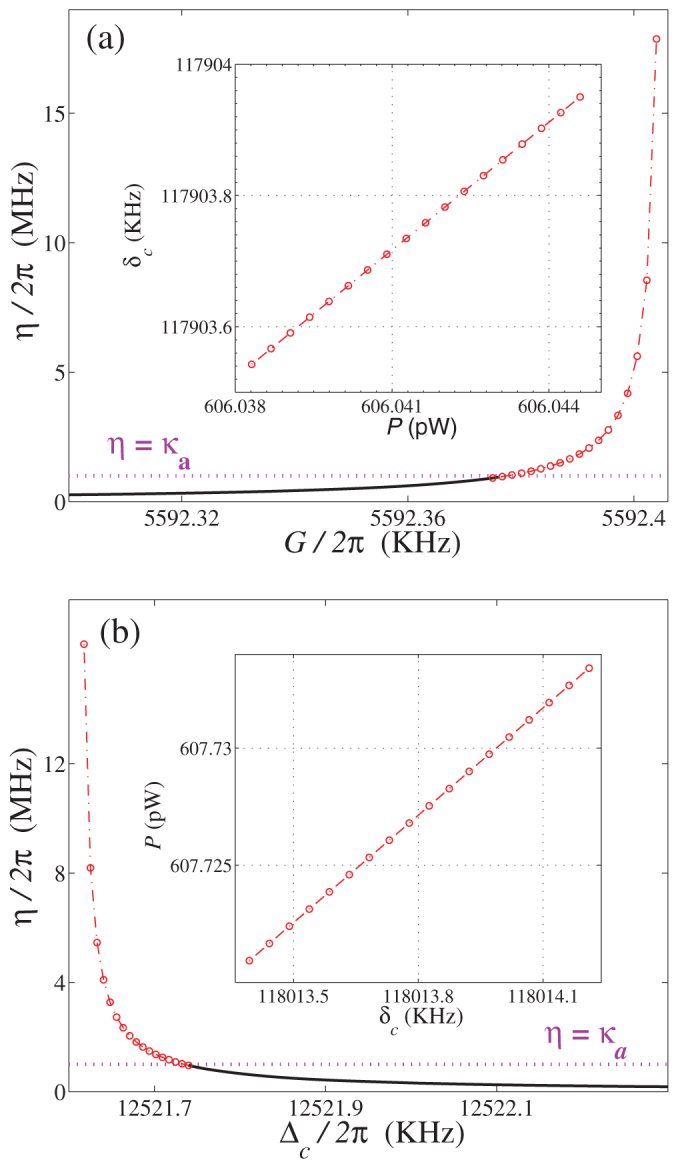
Controllability of the system parameters. Nonlinear parameter *η* versurs: (a) the coupling strength *G* and (b) the effective detuning Δ*_c_*. The inserts present the experimental parameter range (the power *P* and frequency detuning *δ_c_* of the input microwave field) for *η* > *κ_a_*. The system parameters are the same as those in Fig. 2 except for *ω_c_*/2*π* = 7.5 GHz for the inserts.

As one can see, the last two terms in the Hamiltonian (4) show that optical photons can interact with each other through the exchange of the normal modes 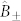, very similar to electrons interacting with each other through the exchange of photons in the QED Hamiltonian. In particular, when the electromechanical subsystem approaches its quantum critical point, the optical cavity shows a strong effective Kerr nonlinearity. This quantum-criticality-induced strong Kerr nonlinearity becomes clear after taking a displacement transformation, 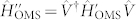, where 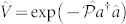 is a similarity transformation and 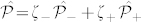 with 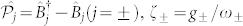. The result is 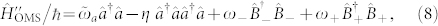
and *η* is the photon-photon interaction strength, 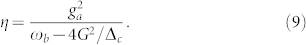
Notice that the photon-photon interaction strength *η* remains unchanged when the system-environment interaction is explicitly included (see the detailed derivation in Methods). On the other hand, it also shows in Figs. 2(c,d) that even in the *weak-coupling regime* 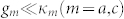, a strong photon-photon interaction *η* (*η* > *κ_a_*) can still be obtained when *G* (or Δ*_c_*) approaches the quantum critical point. In particular, Fig. 2 shows that when the coupling strength *G* (or the detuning Δ*_c_*) is close to its quantum critical point, a very small normal mode frequency *ω*_−_ is obtained, which induces a large photon-photon interaction with *η* ∝ 1/*ω*_−_. The system parameters *G* and Δ*_c_*, determined by the power *P* and the frequency detuning *δ_c_* of the input microwave driving field, can be directly tuned in experiments. Figure 4 shows explicitly the practical parameter range of *P* and *δ_c_* for obtaining the strong Kerr nonlinear parameter *η* (*η* > *κ_a_*).

### Photon blockade

The strong Kerr nonlinearity in the present system can be further demonstrated by the steady-state second-order correlation function of the optical field *g*^(2)^(0). *g*^(2)^(0) → 0 in the weak-coupling regime signals the photon blockade effect, and can be directly detected by a Hanbury-Brown-Twiss Interferometer^3^. Explicitly, by driving the optical cavity with a weak laser field of frequency *ω_ai_* and amplitude *ε_a_*, the Hamiltonian of the system becomes 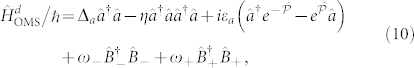
where all the similarity transformations used before have been taken into account, and 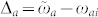. The damping effect arising from the coupling of the optical field to the electromagnetic vacuum modes of the environment can also be taken into account, and the dissipative dynamics of cavity mode 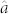 is described by the quantum Langevin equation, 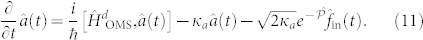
Here *κ_a_* is the decay rate of cavity mode 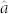 and 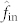 is a vacuum noise operator satisfying 
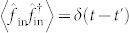
, 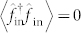.

With a weak optical driving field, the quantum Langevin equation is solved by truncating them to the lowest relevant order in *ε_a_*. The resulting two-photon correlation function is given by 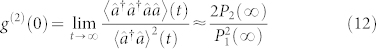
with 
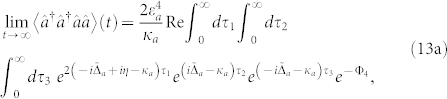

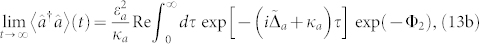
where 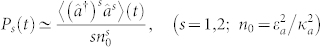
is the normalized *s*-photon probability in the cavity (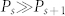 in the weak-driving regime), and 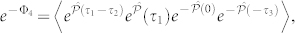

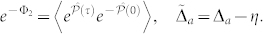


Note that 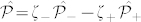 is a complex operator including the microwave field 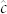 and the mechanical mode 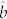, and 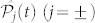 is determined by the dynamics of the electromechanical modes *B_j_*, which evolves as 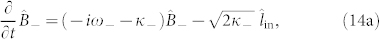

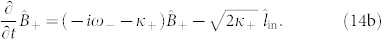


The noise operator 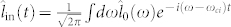, which comes from the environment of the microwave resonator. The environment is initially in the thermal equilibrium state *ρ*_th_ with temperature *T*, and 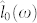 is the initial environment operators of the microwave resonator. Here, we have safely ignored the dissipation of the mechanical oscillator because the mechanical decay rate *κ_b_* is extremely small, *κ_b_*/*κ_a_*, *κ_b_*/*κ_c_* < 10^−3^. Thus, the effective decay rates *κ_j_* is determined by the original decay rate of the microwave resonator *κ_c_* (see the detailed derivation in Methods).

**Figure 5:**
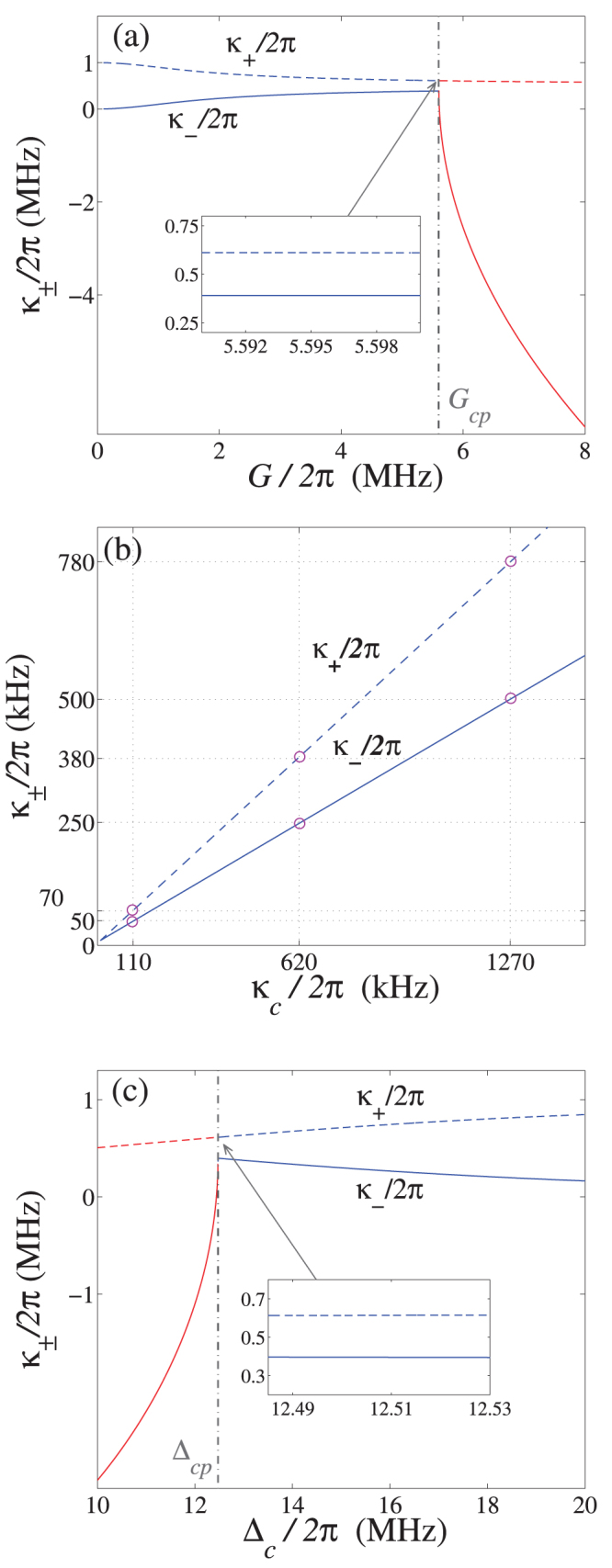
Effective decay rates for the electromechanical normal modes. Effective decay rates *κ*_±_ of the normal modes *ω*_±_ versus: (a) the coupling strength *G*, (b) the decay rate of microwave field *κ_c_*, and (c) the detuning Δ*_c_*. The system parameters are Δ*_c_*/2*π* = 12.51 MHz, *κ_c_*/2*π* = 1 MHz for (a), while *G*/2*π* = 5.5924 MHz, Δ*_c_*/2*π* = 12.51 MHz for (b), and *G*/2*π* = 5.595 MHz, *κ_c_*/2*π* = 1 MHz for (c). The purple circles in (b) indicate the parameter regime corresponding to the *κ*_±_ used in Fig. 6.

In Fig. 5, we show the dependences of *κ*_±_ on the system parameters *G*, Δ*_c_* and *κ_c_*. From Fig. 5 one can see that the effective decay rate *κ*_−_ sharply changes from a positive value to a negative value when the system parameter *G* (or Δ*_c_*) crosses its quantum critical point *G_cp_* (or Δ*_cp_*). This result demonstrates that the mode *ω*_−_ will become a gain mode when *G* > *G_cp_* or Δ*_c_* < Δ*_cp_*. Near the quantum critical points *G_cp_* and Δ*_cp_*, the effective decays *κ*_±_ almost become constant with *G* or Δ*_c_* [see the inserts of Fig. 5(a) and 5(c)]. In Fig. 5(b) *κ*_±_ is plotted via the microwave field decay rate *κ_c_* when *G* (or Δ*_c_*) is near the quantum critical points. As it is shown, *κ*_±_ exhibit a linear increase with the decay rate of the microwave field *κ_c_*.

**Figure 6:**
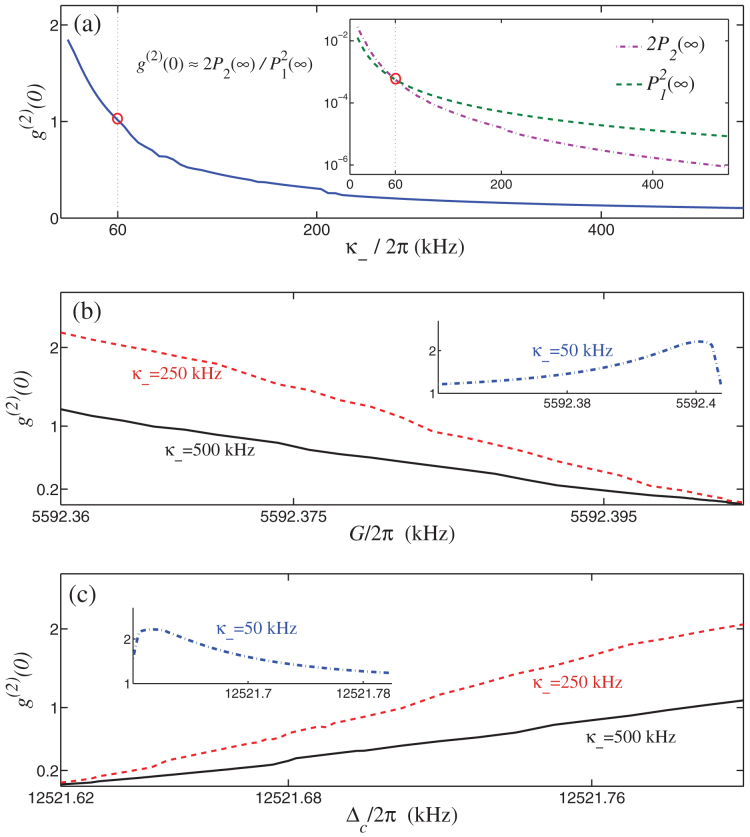
Photon statistics in the hybrid electro-optomechanical system. Equal-time second-order correlation function *g*^(2)^(0) versus: (a) effective decay rate *κ*_−_, (b) coupling strength *G*, and (c) detuning Δ*_c_* at *T* = 0 for the microwave bath. The red circles in (a) indicate the value of *κ*_−_ for *g*^(2)^(0) = 1. We have chosen the quantum critical parameters: *G*/2*π* = 5595 kHz, Δ*_c_*/2*π* = 12521.64 kHz in (a), and the decay rates *κ*_−_/2*π* = (500, 250, 50) kHz (corresponding to *κ_c_*/2*π* = (1270, 620, 110) kHz) in (b,c). The other parameters are the same as in Fig. 2, except for Δ*_a_* = *η* in order to maximize the photon antibunching effect.

When the microwave (mechanical) mode is initially in the coherent state |*α*〉 (|*β*〉), and the optical field in the vacuum state, the two-point correlation function exp(−Φ_2_) and the four-point correlation function exp(−Φ_4_) are calculated. With numerically integrating Eqs. (13), the dependence of *g*^(2)^(0) on *κ*_−_, *G*, and Δ*_c_* is shown in Fig. 6. As one see, in the quantum critical regime, the photon antibunching effect *g*^(2)^(0) < 1 (even the photon blockade *g*^(2)^(0) → 0) occurs because the two-photon transition is largely suppressed in comparison with the single-photon transition when *κ*_−_/2*π* > 60 kHz [see the insert in Fig. 6(a)]. Figures 6(b) and (c) further show that the photon blockade [*g*^(2)^ (0) → 0] occurs when the tunable parameter *G* (or Δ*_c_*) approaches its quantum critical value even if the optomechanical coupling *g_a_* is very weak.

Furthermore, we also find that the photon antibunching [*g*^(2)^(0) < 1] disappears when *κ*_−_/2*π* < 60 kHz [see the inserts in Figs. 6(b) and (c)]. Physically, this is because in the hybrid OMS, a relatively large decay rate *κ*_−_ (*κ*_−_/2*π* > 60 kHz) occurs when the electromechanical subsystem approaches the quantum critical point. This decay will significantly suppress the steadystate sideband transition in the electromechanical subsystem. Then, in the quantum critical regime, the hybrid OMS becomes a pure optical nonlinear system, and *η* > *κ_a_* is the exclusive condition for achieving the photon blockade. Meanwhile, the very small *ω*_−_ (*ω*_−_ → 10 kHz) near the quantum critical point effectively enhances the photon-photon interaction to *η* > *κ_a_* because *η* ∝ 1/*ω*_−_, namely the photon blockade can still be reachable even if the effective electromechanical frequency extends beyond the resolved sideband regime, i.e. *ω*_−_ < *κ_a_*. Notice that the original mechanical frequency used here is still in the resolved sideband regime (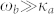) so that there is no problem in cooling the mechanical oscillator at the initial time.

### Nonclassical states

**Figure 7:**
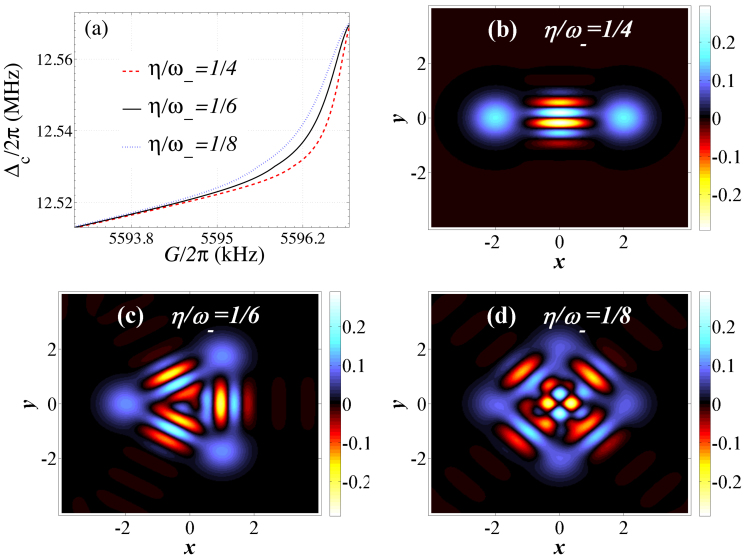
Parameter regimes (a) for obtaining the two- (b), three- (c) and four-component (d) Schrödinger cat state. The quadratures variables are 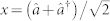, 
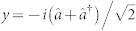
. The system parameters are the same as in Fig. 2 except for 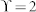.

As demonstrated in previous studies^21^,^22^,^23^, strong Kerr nonlinearities generally lead to the periodic generation of nonclassical states, (e.g., cat states) of the cavity field. With the help of the Hamiltonian (4), we obtain the time evolution operator in the interaction picture, 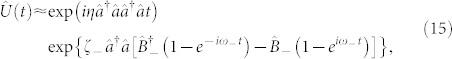
where the term corresponding to *ζ*_+_ has been omitted due to its negligible effect on the evolution of the cavity mode 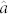 (*ζ*_+_/*ω_b_* ~ 10^−4^) near the quantum critical point. If the cavity field 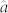 is initially in a coherent state 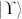, the cavity field at time *t_n_* = 2*nπ*/*ω_−_* (*n* = 1, 2…) will be in the state 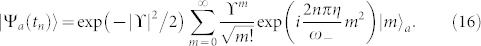
The state |Ψ*_a_*(*t_n_*)〉 is a multi-component cat state, depending on the value of *η*/*ω*_−_. Figure 7 shows the different multi-component cat states for different values of the tunable parameters *G* and Δ*_c_* near the quantum critical point. Figures 7(b,c,d) present the specific realization of two-, three- and four-component cat states, respectively. Here damping effects (given by *κ_a_*, *κ_c_*, *κ_b_*) have been ignored. In principle, this is valid when the cut-off time 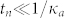, 1/*κ_c_*, 1/*κ_b_*. The optical field damping is similar to that in a recent cavity-QED experiment^54^. Moreover, inspired by Ref. 54, the Wigner function can be measured (or reconstructed) by detecting the states of the atoms interacting with the optical field. Nevertheless, the above result indicates that the quantum-criticality-induced strong Kerr nonlinearities in this hybrid OMS can generate nonclassical states by cutting off the optomechanical interaction at the appropriate time, which can be detected via Wigner tomography.

## Discussion

We have provided a new mechanism for obtaining strong Kerr nonlinear effects in a hybrid OMS in the *weak-coupling regime*. We found that the electromechanical subsystem displays a critical property when adjusting the intensity (or frequency) of the microwave driving field, and a strong controllable photon-photon interaction is induced in the quantum critical regime. Usually, the phonon modulation effect influences the photon statistics in the usual OMSs^24^, and in general will also weaken the photon-photon interaction effect, except in the single-photon strong-coupling (*g_a_* > *κ_a_*) and the resolved sideband (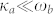) regime^24^. The essence of the strong photon-photon interaction presented in this paper can be understood as follows. In the quantum critical regime, the electromechanical normal mode 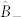 coupled to the optical field is highly softened (or a very small normal-mode frequency *ω*_−_ is obtained). At the same time, the sideband phonon transitions in the electromechanical subsystem are significantly suppressed by the relative large decay rate of the electromechanical normal mode, which makes the hybrid OMS essentially a pure optical nonlinear system. Thus, the quantum-criticality-induced strong self-Kerr nonlinearity is very different from previous investigations in the usual OMSs^24^,^34^,^35^.

Experimentally, the strong photon-photon interaction achieved in the present hybrid OMS requires driving the electromechanical subsystem into its quantum critical region (shaded area in Fig. 2). Normal-mode splitting in the driven electromechanical system has been observed^43^. The quantum critical region could be easily reached by increasing the intensity of the microwave driving field. Moreover, as shown in Figs. 2 and 4, the interesting ranges of *G* and Δ*_c_* are respectively on the order of 0.1 kHz and 1 kHz for the quantum critical region, and this parameter precision is experimentally realizable^55^. We believe that our proposal will provide a new avenue for experimentally realizing strong optical nonlinearities in the *weak-coupling regime* and largely enrich the scope of implementing quantum information processing and quantum metrology with cavity OMSs.

## Methods

### Derivation of the photon-photon interaction with system-environment couplings

The total Hamiltonian of the hybrid OMS plus the environment can be written as 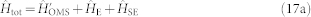
where the system Hamiltonian 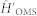 is given by Eq. (4) and 
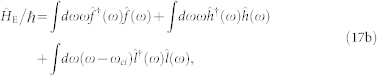

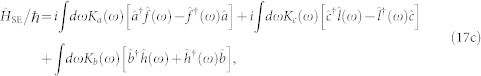
are the Hamiltonians of the environment and the system-environment interaction, respectively. Notice that the system-environment interaction is invariant to the linearization procedure applied on the electromechanical subsystem. Here 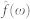, 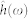, 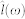 are the bath operators for 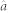, 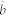, 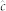, and *K_j_*(*ω*) (*j* = *a*, *b*, *c*) are the corresponding coupling constants. For a slowly-varying bath spectrum, we can simply replace *K_j_*(*ω*) by the decay rate 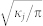. Here the last term can be safely neglected because the decay rate *κ_b_* of the mechanical oscillator is extremely small (*κ_b_*/*κ_a_*, *κ_b_*/*κ_c_* < 10^−3^).

By applying a Bogoliubov transformation 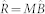 to the total Hamiltonian 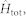, the hybrid OMS Hamiltonian 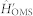 and the interaction between the system and the environment 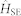 can be rewritten in terms of the normal-mode canonical operators 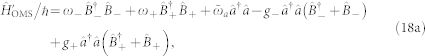

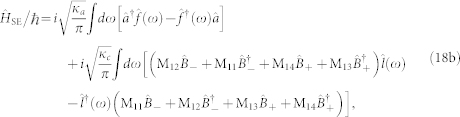
while the environment Hamiltonian 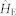 retains its original form.

To derive the photon-photon interaction, the total Hamiltonian should be further diagonalized in a displaced-oscillator representation, 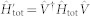, and the result is 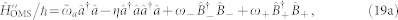

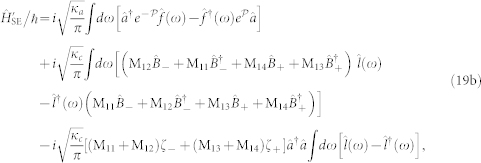
where 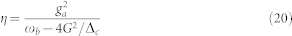
is the photon-photon interaction strength. This similarity transformation also does not affect the environment Hamiltonian 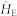. By comparing with the dissipation Hamiltonian in the original representation [Eq. (18b)], it can bee seen that the last term of Eq. (19b) is induced by the similarity transformation in the displaced-oscillator representation, and it may change the photon-photon interaction. However, we will show next that, in the quantum critical regime, this term will not change the photon-photon interaction *η*, and it only induces a negligible pure-dephase of the optical mode.

In the quantum critical regime, the system parameters M_13_, M_14_, and *ζ*_+_ are negligible compared to the parameters M_11_, M_12_, and *ζ*_−_, due to the relative large frequency *ω*_+_ (M_13_, M_14_, and *ζ*_+_ are smaller than M_11_, M_12_, and *ζ*_−_ by about 3 to 4 orders of magnitude). This means that the influence of the normal mode *B*_+_ on the dynamics of the optical mode 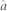 can be safely neglected when the electromechanical subsystem approaches its quantum critical point. By ignoring the normal modes 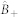, the dynamics of the bath operator 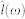 can be determined by the following equation of motion, 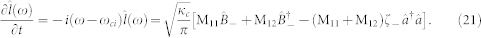
Solving Eq. (21), the result is 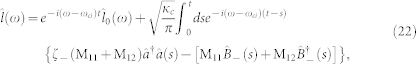
where 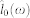 is the initial environment operator of the microwave resonator. Substituting the above solution of the bath operator and its hermitian conjugate into the last term of Eq. (19b) and noticing that in the quantum-critical-regime 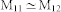, we have 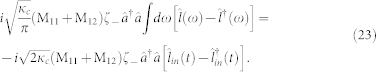
Therefore, the photon-photon interaction given in Eq. (19a) remains invariant under the interaction with the environment.

### Calculation of the effective decay rates for the electromechanical normal modes

Based on the dissipative dynamics of the electro-mechanical subsystem in the original representation, we can find the relationship between the effective decay rates *κ*_±_ and the original decay rates of the microwave resonator *κ_c_*.

Considering the thermal environments of the microwave resonator, the Hamiltonian of the electro-mechanical subsystem plus the environment is 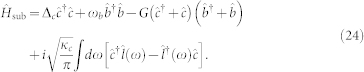
Then, the dynamics of the canonical operator 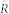 is given by 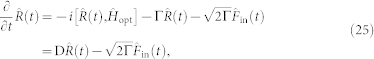
where the coefficient matrix 
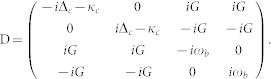


Here, Γ = diag(*κ_c_*, *κ_c_*, 0, 0) denotes the decay rates of the microwave resonator and the mechanical oscillator, and 
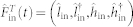
 are the Langevin forces. Equation (25) shows that the imaginary and real parts of the eigenvalues of D correspond to the eigenfrequencies *ω*_±_ and the effective decay rates *κ*_±_ of the normal modes. For the undamped case (*κ_c_* = 0), the eigenvalues of D are purely imaginary and we obtain the expression Eq. (5) for the normal-mode frequencies. For the general *κ_c_*, we numerically diagonalized the coefficient matrix D and shown the results in Fig. 5.

## Author Contributions

X.Y.L. carried out all calculations under the guidance of W.M.Z. and S.A., Y.W. and F.N. participated in the discussions. All authors contributed to the interpretation of the work and the writing of the manuscript.
